# Reprogramming of *Escherichia coli* K-12 Metabolism during the Initial Phase of Transition from an Anaerobic to a Micro-Aerobic Environment

**DOI:** 10.1371/journal.pone.0025501

**Published:** 2011-09-27

**Authors:** Eleanor W. Trotter, Matthew D. Rolfe, Andrea M. Hounslow, C. Jeremy Craven, Michael P. Williamson, Guido Sanguinetti, Robert K. Poole, Jeffrey Green

**Affiliations:** 1 Department of Molecular Biology and Biotechnology, The Krebs Institute, University of Sheffield, Sheffield, United Kingdom; 2 Informatics Forum, University of Edinburgh, Edinburgh, United Kingdom; East Carolina University School of Medicine, United States of America

## Abstract

**Background:**

Many bacteria undergo transitions between environments with differing O_2_ availabilities as part of their natural lifestyles and during biotechnological processes. However, the dynamics of adaptation when bacteria experience changes in O_2_ availability are understudied. The model bacterium and facultative anaerobe *Escherichia coli* K-12 provides an ideal system for exploring this process.

**Methods and Findings:**

Time-resolved transcript profiles of *E. coli* K-12 during the initial phase of transition from anaerobic to micro-aerobic conditions revealed a reprogramming of gene expression consistent with a switch from fermentative to respiratory metabolism. The changes in transcript abundance were matched by changes in the abundances of selected central metabolic proteins. A probabilistic state space model was used to infer the activities of two key regulators, FNR (O_2_ sensing) and PdhR (pyruvate sensing). The model implied that both regulators were rapidly inactivated during the transition from an anaerobic to a micro-aerobic environment. Analysis of the external metabolome and protein levels suggested that the cultures transit through different physiological states during the process of adaptation, characterized by the rapid inactivation of pyruvate formate-lyase (PFL), a slower induction of pyruvate dehydrogenase complex (PDHC) activity and transient excretion of pyruvate, consistent with the predicted inactivation of PdhR and FNR.

**Conclusion:**

Perturbation of anaerobic steady-state cultures by introduction of a limited supply of O_2_ combined with time-resolved transcript, protein and metabolite profiling, and probabilistic modeling has revealed that pyruvate (sensed by PdhR) is a key metabolic signal in coordinating the reprogramming of *E. coli* K-12 gene expression by working alongside the O_2_ sensor FNR during transition from anaerobic to micro-aerobic conditions.

## Introduction

*Escherichia coli* is a metabolically versatile bacterium that experiences transitions between aerobic (outside a host) and anaerobic (in the lower intestine of a host) niches as part of its lifestyle. Therefore, the ability to adapt to environments with different O_2_ availabilities is vital for *E. coli* competitiveness in Nature and requires reprogramming of gene expression.

**Figure 1:**
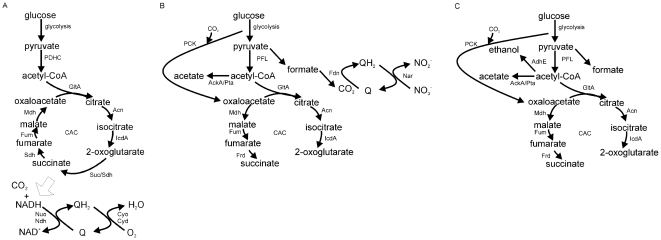
The metabolic modes of *Escherichia coli*. The main pathways of carbon and energy metabolism are shown for (A) aerobic respiration, (B) anaerobic (nitrate) respiration, and (C) fermentation. Under aerobic conditions the citric acid cycle (CAC) is functional, whereas under anaerobic conditions the non-cyclic form operates. Under aerobic conditions glucose can be completely oxidized to CO_2_ and water. Under anaerobic conditions in the presence of an alternative electron acceptor such as nitrate, glucose is partially oxidized to CO_2_ and acetate. Under anaerobic fermentative conditions glucose is converted to acetate, ethanol and formate and energy is conserved by substrate-level phosphorylation rather than oxidative phosphorylation. The enzyme activities responsible for each step are indicated: AckA, acetate kinase; Acn, aconitase B; AdhE, alcohol dehydrogenase; Cyd, cytochrome *bd* oxidase; Cyo, cytochrome *bo* oxidase; Fdn, formate dehydrogenase-N; Fum, fumarase (A, B and C); GltA, citrate synthase; IcdA, isocitrate dehydrogenase; Mdh, malate dehydrogenase; Nar, nitrate reductase; Nuo, NADH dehydrogenase I; Ndh, NADH dehydrogenase II; PCK, phosphoenolpyruvate carboxykinase; PDHC, pyruvate dehydrogenase complex; PFL, pyruvate formate-lyase; Pta, phosphotransacetylase; Sdh, succinate dehydrogenase; Suc, 2-oxoglutarate dehydrogenase/succinyl-CoA synthetase.

*Escherichia coli* has three basic metabolic modes that depend on the availabilities of combinations of electron donors and electron acceptors [1]–[3]. When O_2_ is available, aerobic respiration allows the complete oxidation of a growth substrate, such as glucose, with the maximum potential to conserve energy (Figure 1A). Aerobic respiration is therefore the most productive and the preferred metabolic mode [1]. During aerobic metabolism, the pyruvate produced by glycolysis is oxidatively decarboxylated by the pyruvate dehydrogenase complex (PDHC) to yield CO_2_, NADH, and acetyl-CoA. Oxidation of the acetyl units by the citric acid cycle (CAC) generates reducing equivalents (e.g. NADH) that are used in the reduction of O_2_ to H_2_O via aerobic electron transport chains, thereby creating proton gradients that can be used to generate ATP and drive other essential activities (Figure 1A). In the absence of O_2_, two alternative metabolic modes are possible. If an alternative terminal electron acceptor, such as NO_3_^−^, is available anaerobic respiration is possible (Figure 1B). During anaerobic respiration the role of the PDHC is assumed by pyruvate formate-lyase (PFL), the CAC is repressed with the metabolic flow in the C_4_ section of the CAC reversed, offering the possibility of some energy conservation by the reduction of fumarate to succinate by fumarate reductase (Figure 1B) [4]. The energy conserved by anaerobic respiration is less than that achieved by aerobic respiration because the substrate, glucose, is only partially oxidized, with acetate being excreted as a major reduced end product (overflow metabolite) [1]. However, anaerobic respiration is more productive than fermentation, where energy is conserved by substrate-level phosphorylation, and redox balance is achieved by the formation of the overflow metabolites, acetate, ethanol, formate and succinate (Figure 1C) [5]. Thus, O_2_ availability has profound effects on *E. coli* physiology that are mediated by altered patterns of gene expression which determine the metabolic fate of pyruvate.

One of the key regulators of pyruvate fate is the transcription factor PdhR, which represses the expression of genes encoding the PDHC [6], and the PFL-repair protein, YfiD [7]. When PdhR interacts with pyruvate it no longer binds DNA and the expression of its target genes is derepressed [7], [8]. Expression of the *pdh-aceEF-lpdA* operon and *yfiD*, as well as some other PdhR-regulated genes, is also controlled by the O_2_-sensing transcription factor FNR [9]–[12]. Thus FNR and PdhR work together to integrate responses to an environmental (O_2_) and a metabolic (pyruvate) signal to reprogram gene expression and optimize metabolism.

Most reports of the effects of O_2_ on transcription in *E. coli* have compared separate aerobic and anaerobic cultures often grown in batch culture and in complex medium [10], [12]–[14]. Thus, there is relatively little information on transcriptome dynamics during adaptation to changes in O_2_ availability. The transcriptional responses that occur when steady-state anaerobic cultures of *E. coli* are exposed to air to create fully aerobic conditions have been reported [15]. It was shown that within 5 min the abundances of transcripts associated with anaerobic metabolism (anaerobic reductases and hydrogenases) were decreased, whilst transcripts associated with aerobic metabolism (PDHC, CAC and aerobic electron transport chains) and the peroxide stress response were increased [15]. Here, for the first time, the dynamics of adaptation when anaerobic *E. coli* K-12 cultures are transferred to micro-aerobic conditions are reported. The approach is based on perturbation of anaerobic steady-state chemostat cultures by introducing O_2_ to create micro-aerobic conditions whilst keeping all other parameters constant. The process of adaptation to the new environment was followed using time-resolved transcript profiling, inference of transcription factor activities, protein abundance measurements, and analysis of metabolite profiles. The data obtained are consistent with rapid O_2_-mediated inactivation of FNR and PFL activities, causing pyruvate production to exceed the capacity for its utilization, which in turn inactivates PdhR, resulting in the reprogramming of gene expression to facilitate the switch from fermentative to micro-aerobic respiratory metabolism.

## Results and Discussion

Anaerobic steady-state cultures of *E. coli* K-12 MG1655 in glucose-limited minimal medium were established in the chemostat and then perturbed by the introduction of air to maintain a dissolved O_2_ concentration in the medium of 10 µM. Samples were removed after 5, 10, 30, 60 and 120 min to measure the abundances of transcripts, some key proteins and extra-cellular metabolites. A shift to 10 µM O_2_ was chosen following measurements of dissolved O_2_ concentrations in steady-state cultures across the aerobiosis scale as defined by Alexeeva *et al*. [13], which showed that ∼10 µM dissolved O_2_ was detectable at 85% aerobiosis. The aerobiosis scale applies to the carbon-limited cultures used here and 100% aerobiosis is defined as the minimum O_2_ input that results in undetectable acetate production i.e. full aerobic respiratory growth. Decreasing O_2_ supply linearly increases the specific rate of acetate production to a maximum under anaerobic conditions, which is defined as 0% aerobiosis. Micro-aerobic conditions, i.e. between 0 and 100% aerobiosis, are characterized by intermediate specific acetate production rates [13].

### Transcript profiles indicate changes in central metabolism upon transfer to micro-aerobic growth

The introduction of air to the anaerobic steady-state cultures resulted in predictable changes in the abundances of transcripts encoding proteins of central metabolism, including induction of transcripts encoding: the PDHC, aconitase B, the 2-oxoglutarate dehydrogenase complex, succinate dehydrogenase, NADH dehydrogenase II, the cytochrome *bo* terminal oxidase, and fumarase C (Table 1). Furthermore, transcripts associated with anaerobic metabolism such as those that encode PFL, hydrogenase proteins, aspartase, alcohol dehydrogenase, terminal reductases and cytochrome *bd* II oxidase (*appBC*) were less abundant after perturbation of the anaerobic steady-state (Table 1). The PFL repair protein YfiD was induced indicating that the cultures were experiencing micro-aerobic conditions (Table 1) [11], [16]. Furthermore, the specific rate of acetate production for the anaerobic steady-state was ∼8.2 mmoles h^−1^ g^−1^ cdw (cell dry weight) compared to ∼6.2 mmoles h^−1^ g^−1^ cdw for the 60–120 min period of the transition i.e. after transition acetate production was sub-maximal but non-zero, indicative of micro-aerobic growth.

**Table 1:** Transcripts encoding proteins of central metabolism that are present in altered abundance after shifting anaerobic cultures of *E. coli* MG1655 to micro-aerobic (10 µM O_2_) conditions.

*Operon name	Product	†Fold change in abundance relative to the anaerobic steady-state at the indicated times (min) following the switch to micro-aerobic conditions					‡Relevant regulatory proteins
		5	10	30	60	120	
*acnB*	aconitase B	2.3	4.1	3.1	3.3		ArcA (−)
*adhE*	alcohol dehydrogenase	0.43	0.27	0.42	0.24	0.32	FNR (+)
*appBC*	cytochrome *bd*II				0.30	0.24	AppY (+) ArcA (+)
*aspA*	aspartase	0.37	0.26	0.22	0.19	0.21	FNR (+)
*cyoA*-*E*	cytochrome *bo* oxidase	25.1	27.2	29.6	31.1	29.6	ArcA (−) FNR (−) PdhR (−)
*fdoGHI-fdhE*	formate dehydrogenase	4.3	3.8		4.7		
*focA-pflB*	formate transport/PFL	0.50	0.42	0.48	0.4		ArcA (+/−) FNR (+)
*frdABCD*	fumarate reductase				0.44		FNR (+)
*fumC*	fumarase	2.1	3.8	3.2	4.0		ArcA (−)
*gltA*	citrate synthase				0.27		ArcA (−)
*hyaA-F*	hydrogenase 1				0.21	0.14	ArcA (+)
*hycA-I*	hydrogenase 3			0.42	0.19	0.06	
*hypA-E*	hydrogenase 3 accessory proteins			0.4	0.33	0.26	FNR (+)
*narGHIJ*	nitrate reductase	0.48	0.13	0.22	0.14	0.29	FNR (+)
*ndh*	NADH dehydrogenase II	3.9	5.7	7.5	5.4	5.3	ArcA (−) FNR (−) PdhR (−)
*nirB-D-cysG*	nitrite reductase	0.36	0.23	0.2	0.11	0.13	FNR (+)
*nrfA-F*	nitrite reductase	0.33	0.13	0.08	0.05	0.06	FNR (+)
*pdhR-aceEF-lpdA*	PDHC	4.0	4.5	4.0	3.5	2.7	FNR (−/+) PdhR (−)
*sdhCDAB*	succinate dehydrogenase	13.4	12.1	3.8	2.2		ArcA (−/+) FNR (−)
*sucABCD*	succinyl-CoA synthetase		4.1				
*yfiD*	PFL repair protein	7.1	9.2	12.0	6.5	5.1	ArcA (+) FNR (+/−) PdhR (−)

*The data shown are for the first gene in the operon.

† Fold change (by at least 2-fold at one or more time-points) is the ratio of transcript levels at the indicated time to the transcript levels at t = 0 (the initial anaerobic state; *p*≤0.05).

‡ Relevant regulatory proteins are indicated (−) denotes negative regulation, (+) positive regulation, and (−/+) negative andtab positive regulation. The Table was compiled from references in the text and EcoCyc database [17]. Cells with no entry indicate no significant change in transcript abundance.

### The abundances of central metabolic enzymes correlate with changes in the abundances of the corresponding transcripts

**Figure 2:**
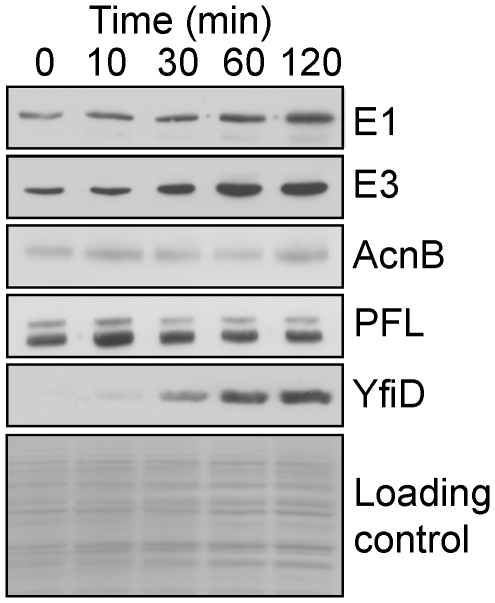
Western blot showing the induction of selected central metabolic proteins after transfer of anaerobic cultures to micro-aerobic conditions. Anaerobic steady-state fermentative cultures were perturbed by introduction of air to maintain a dissolved O_2_ tension of 10 µM. Samples were taken before and after perturbation at the indicated times. The samples were analyzed by Western blotting using polyclonal antibodies to the PDHC components E1 and E3, aconitase B (AcnB), pyruvate formate-lyase (PFL), and PFL repair protein (YfiD). Total protein loading in each track is shown as the Coomassie blue-stained gel in the bottom panel.

Confirmation that the changes in transcript profiles (Table 1) were physiologically relevant was obtained by the generally good correlations between the abundances of PDHC (E1 and E3 components), aconitase B, PFL and YfiD proteins as judged by Western blotting (Figure 2), and the changes in abundance of the corresponding transcripts (Table 1). Measurement of PDHC activity (Table 2) was also consistent with the abundances of E1 and E3 and *pdhR-aceEF-lpd* transcript.

**Table 2:** Pyruvate dehydrogenase complex activity during transition from anaerobic to micro-aerobic conditions.

Time after aeration (min)	PDHC activity (mmole h^−1^ g^−1^ cdw)
0	0.6
10	1.1
30	1.4
60	1.8
120	2.0

Anaerobic steady-state chemostat cultures were sampled for measurement of PDHC activity before and after transfer from anaerobic to micro-aerobic (10 µM O_2_) conditions. Cell-free extracts were prepared and PDHC activity was measured as described in the *Methods and Materials* section. Data are means from two experiments that varied by <10%. Protein concentration was converted to cdw on the basis that *E. coli* cdw is 65% protein [18].

### The transition from anaerobic to micro-aerobic growth causes transient excretion of pyruvate

**Figure 3:**
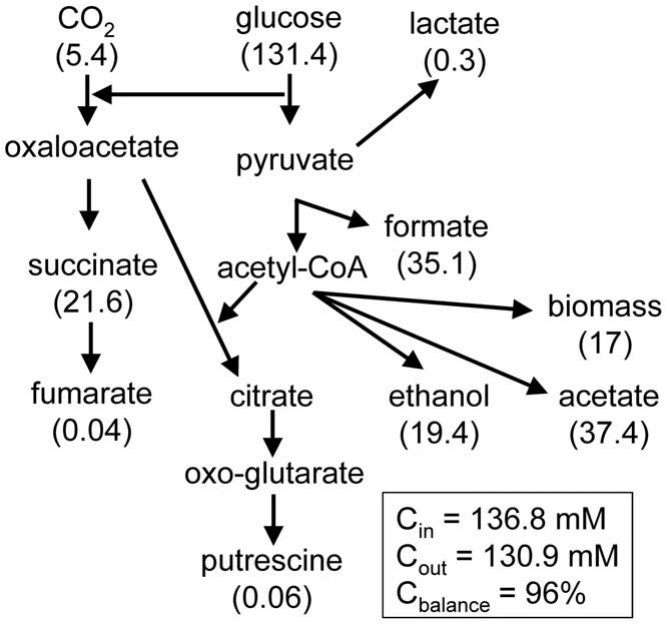
Carbon balance for the anaerobic steady-state cultures. The carbon input into the system comes from glucose and carbon dioxide supplied to the culture (C_in_). Carbon output consists of overflow metabolite production and biomass (C_out_). At steady-state C_in_ = C_out_ (i.e. C_balance_ = 100%). The numbers in parentheses are calculated from the values in Table 3 and are the average (n = 4) concentrations of C (mM) in each of the indicated species as measured by NMR.

Measurement of the extra-cellular metabolome of the anaerobic steady-state cultures by ^1^H-NMR showed the presence of acetate (∼18.7 mM), ethanol (∼9.7 mM), formate (∼35.1 mM), lactate (∼0.1 mM) and succinate (∼5.4 mM), confirming that the cultures were glucose-limited (21.9 mM input, undetectable residual) and growing (cell dry weight, cdw = 0.46 mg ml^−1^, 44.3% cdw is C; [18]) by fermentation (Table 3; Figure 3). The calculated carbon balance based on the average values of biomass and extra-cellular metabolite concentrations was 96%, providing confidence in the accuracy of the NMR-based metabolite concentration measurements.

**Table 3:** Measurements of extra-cellular metabolites and biomass production during transition of anaerobic cultures of *E. coli* MG1655 to micro-aerobic conditions.

Time after transition to micro-aerobic (10 µM O_2_) conditions (min)	Acetate (mM)	Formate (mM)	Succinate (mM)	Putrescine (mM)	Pyruvate (mM)	Lactate (mM)	Fumarate (mM)	Ethanol (mM)	Biomass (mg cdw ml^−1^)
0	18.7±2.2	35.1±4.0	5.3±0.9	0.015±0.003	<0.10	0.12±0.03	0.01±0.007	9.7±1.1	0.46±0.04
10	17.7±1.1	32.9±1.5	4.8±0.6	0.023±0.001	0.41±0.10	0.14±0.02	0.07±0.02	9.1±0.4	0.47±0.04
30	16.8±1.2	30.9±2.1	4.3±0.5	0.042±0.013	0.90±0.27	0.18±0.02	0.18±0.02	8.5±0.5	0.52±0.06
60	15.3±1.1	26.6±2.1	3.4±0.3	0.043±0.003	0.99±0.39	0.18±0.04	0.25±0.04	6.9±1.0	0.54±0.07
120	16.6±0.7	22.6±0.9	2.3±0.1	0.055±0.010	0.23±0.31	0.14±0.03	0.32±0.04	6.5±0.7	0.65±0.06

Metabolites were quantified by NMR as described in *Materials and Methods* section. The values are the means from four independent cultures ±standard deviation. The errors for the pyruvate data were relative large and therefore a t-test analysis was performed which showed the 0–10 min (*p* = 0.053), the 10–30 min (*p* = 0.004), 60–120 min (*p* = 0.009) but not the 30–60 min (*p* = 0.828) were significantly different.

After the introduction of O_2_, the production of anaerobic overflow metabolites was altered such that the amounts of formate, ethanol and succinate decreased as would be expected if production ceased upon exposure to O_2_ i.e. equivalent to the dilution rate of the culture (0.2 h^−1^), consistent with inhibition of PFL and fumarate reductase activity accompanying a switch to aerobic metabolism (Table 3). However, acetate production was maintained indicating that complete conversion to fully aerobic respiratory growth was not achieved within the first 120 min of adaptation (Table 3) because medium from cultures growing by aerobic respiration do not contain acetate under the conditions used here [19] . Moreover, the extra-cellular concentration of fumarate increased during the transition, suggesting that fumarase activity was a bottleneck in the CAC, even though the *fumC* transcript was significantly induced (Table 1).

A striking feature of the overflow metabolite profile was the transient excretion of pyruvate by the cultures (Table 3). Pyruvate was undetectable (<0.1 mM) in the anaerobic steady-state (Table 3). The rate of pyruvate excretion was maximal in the 0–10 min interval and decreased thereafter until in the 60–120 min interval there was net consumption of excreted pyruvate (the decrease in extra-cellular pyruvate concentration was greater than that accounted for by the dilution rate of the culture). These changes in pyruvate excretion are a useful indicator of the physiological changes occurring during adaptation to micro-aerobic conditions (Table 3). Pyruvate stands at a major junction in *E. coli* K-12 aerobic-anaerobic metabolism (Figure 1). Under aerobic conditions the pyruvate is oxidized to acetyl-CoA and CO_2_ by the PDHC (Figure 1A), whereas under anaerobic conditions pyruvate is converted to acetyl-CoA and formate by the action of PFL (Figure 1C) [1]. The observed transient excretion suggests that the production of pyruvate temporarily exceeds the bacteria's capacity to metabolize pyruvate after transfer to micro-aerobic conditions. The simplest explanation to account for the transient excretion of pyruvate is that a sustained glycolytic flux was accompanied by inactivation of PFL before PDHC activity was sufficient to compensate for the loss of PFL activity. Accordingly, the transcript profiles, Western blots of the E1 and E3 components of the PDHC, and measurement of PDHC activities indicated that maximum PDHC activity was only achieved 60–120 min after aeration (Table 2; Figure 2).

### Pyruvate- (PdhR) and O_2_- (FNR) responsive transcription factors are inactivated during the transition from anaerobic to micro-aerobic conditions

**Figure 4:**
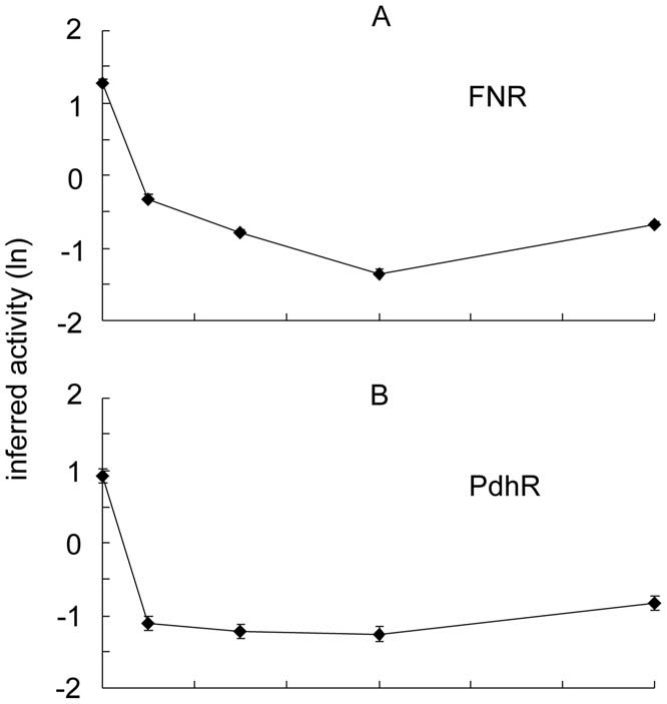
Inferred activity of FNR and PdhR. The inferred activities of the transcription factors FNR (A) and PdhR (B) are shown as natural log (ln) values. The error bars (mostly within the size of the symbols) represent the standard deviation provided by the posterior distribution.

One of the key regulators of pyruvate fate is the transcription factor PdhR, which represses expression of the PDHC, the PFL-repair protein YfiD, NADH dehydrogenase II, and the cytochrome *bo'* oxidase (Cyo); all these transcripts exhibit altered abundance during the transition to micro-aerobic growth (Table 1). When PdhR interacts with pyruvate it no longer binds DNA and the expression of target genes is derepressed [6], [8]. Expression of the *pdh* operon, *yfiD*, *ndh*, and the *cyo* operon is also regulated by the O_2_-sensing transcription factor FNR [9]–[12]. Therefore the time-resolved transcriptomic datasets described above were analyzed using a probabilistic state space model [20] to infer the activities of PdhR and FNR during adaptation to micro-aerobic conditions. The model implied that FNR activity rapidly decreases upon exposure of cultures to micro-aerobic conditions reaching a minimum of ∼15-fold lower than the anaerobic activity after 60 min and recovering to be ∼7-fold lower than the anaerobic activity thereafter (Figure 4A). This pattern of FNR activity suggests that, after 60 min aerobic respiratory activity at the cell membrane consumes sufficient O_2_ to lower O_2_ concentrations in the cytoplasm with consequent restoration of some FNR activity. Consistent with previous reports which showed that the abundance of the *cydAB* transcript, encoding cytochrome *bd*-I terminal oxidase that has high affinity for O_2_, was only enhanced when the O_2_ supply was cut off from aerobic cultures but not when it was supplied to anaerobic cultures [15], [21], was not significantly altered here. This suggests that O_2_ consumption at the membrane would initially occur by the action of pre-existing cytochrome *bd*-I terminal oxidase, which is known to present in anaerobic cultures [19]. Synthesis of the cytochrome *bo* terminal oxidase (as indicated by increased transcript abundance; Table 1) would then supplement the rate of O_2_ consumption later in the transition facilitating the restoration of some FNR activity. Similarly, the ability of PdhR to bind DNA and repress transcription was predicted to decrease (∼10-fold less active) to reach a minimum between 10–60 min of exposure to O_2_, before recovering at the 120 min time-point to reach an activity level ∼6-fold lower than that predicted for anaerobic conditions (Figure 4B). This pattern of PdhR activity is consistent with the proposal that upon exposure of cultures to O_2_ the production of pyruvate by the bacteria exceeds the capacity to metabolize this key metabolite.

### Conclusion

**Figure 5:**
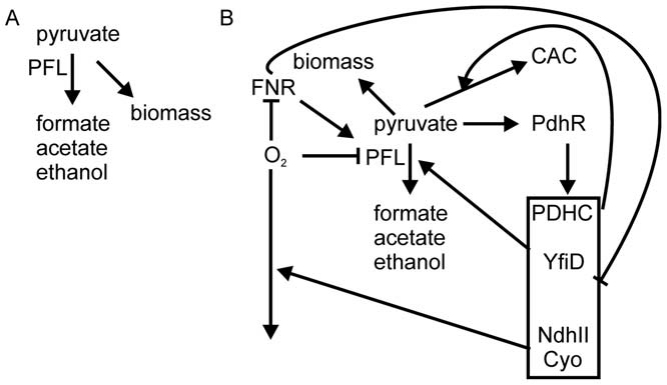
Model illustrating the role of pyruvate as a secondary messenger in managing the transition of *E. coli* K-12 from anaerobic to micro-aerobic conditions. In the absence of O_2_ (A) carbon (pyruvate) is incorporated into biomass or excreted as overflow metabolites (mostly formate, acetate and ethanol) in a process initiated by pyruvate formate-lyase (PFL). Upon transfer to micro-aerobic conditions (B) O_2_ inactivates PFL, directly by oxygenolytic cleavage, indirectly through the action of PFL deactivase (AdhE) [23]. Inactivation of the O_2_-sensing transcription factor FNR inhibits expression from promoter 6 of *focA-pflB* [24]. Inhibition of PFL activity at transcriptional and protein levels and a delay in induction of pyruvate dehydrogenase complex (PDHC) activity (Tables 1 and 2) results in a deficit in the capacity to metabolize the pyruvate generated by glycolysis. This hiatus in pyruvate metabolism is sufficient to inactivate the transcription factor PdhR. Inactivation of PdhR derepresses expression of genes encoding the PDHC and the PFL repair protein (YfiD). This response acts to restore the capacity to metabolize pyruvate and as a result extra-cellular pyruvate concentration begins to decline 60 min into the transition. In addition, pyruvate-mediated derepression of the PdhR-repressed genes encoding the primary dehydrogenase NdhII and the terminal oxidase cytochrome *bo'* oxidase (Cyo) [8] increases the consumption of O_2_ at the cell membrane, potentially stabilizing undamaged/newly activated cytoplasmic PFL activity and restoring some activity to the O_2_-sensing transcription factor, FNR, promoting limited anaerobic metabolism (e.g. acetate excretion, Table 3).

The approach adopted here has provided new insight into the dynamics of transcriptional, metabolic and physiological adaptations of *E. coli* K-12 during the initial phases of transition from an anaerobic to a micro-aerobic environment. The data suggest that introducing O_2_ into the system results in the immediate inhibition of PFL (as indicated by the cessation of formate production) before PDHC and repaired/newly synthesized PFL activities are sufficient to process the pyruvate produced by glycolysis. This hiatus in metabolism results in the reprogramming of gene expression through the action of the pyruvate sensor PdhR in concert with the O_2_ sensor FNR. The consequences of the transcriptional reprogramming includes the synthesis of the PFL repair protein YfiD, NADH dehydrogenase II and cytochrome *bo'* oxidase to initiate aerobic respiration (Figure 5). The inferred FNR activity pattern suggests that after 60 min of O_2_ exposure aerobic respiratory activity at the cell membrane consumes sufficient O_2_ to lower O_2_ concentrations in the cytoplasm thereby supporting some FNR activity and protecting any repaired/newly synthesized PFL activity. The inferred PdhR activity implies that it is also rapidly inactivated. The consequent derepression of the PDHC gradually increases the capacity to utilize pyruvate, such that in the 60–120 min interval previously excreted pyruvate is consumed and PdhR activity is partially restored. Thus, the transition from anaerobic to micro-aerobic conditions requires a complex network of transcriptional and metabolic adaptations that are, at least in part, coordinated by FNR and PdhR in response to environmental and metabolic signals (Figure 5).

## Materials and Methods

### Bacteria and growth conditions

Chemostat cultures *E. coli* strain MG1655 of were grown in a 1 l capacity Labfors 3 chemostat (Infors) at 37°C and with stirring at 400 rpm. The pH was maintained at 7.0 by automatic titration with sterile 1 M KOH or 1 M H_2_SO_4_. Carbon-limited Evans defined medium was used with 21.9 mM glucose as the carbon source [22]. After inoculation with 5 ml overnight culture, the cells were grown in the chemostat overnight as a batch culture. Steady state was then achieved by feeding growth medium at a dilution rate of 0.2 h^−1^. Anaerobic cultures were maintained by sparging with N_2_ (95%) and CO_2_ (5%) at 0.4 l min^−1.^ Dissolved O_2_ levels were monitored using a TruDO Dissolved Oxygen Sensor (Finesse). The switch to micro-aerobic conditions was achieved by automatically altering the gas mix ratio of N_2_ and CO_2_ to air to maintain the dissolved O_2_ tension in the culture at 5% (∼10 µM).

### Transcript profiling

At the times indicated, samples were removed from the chemostat directly into *RNA protect* before purification using an RNeasy mini kit (Qiagen) with on-column DNase I treatment according to the manufacturer's instructions. The integrity of the RNA was determined by gel electrophoresis and concentration was determined spectrophotometrically. RNA (10 µg) from control (anaerobic steady state culture) or experimental samples (5, 10, 30, 60 or 120 min after shift to 10 µM O_2_) was used to prepare Cy3-dCTP or Cy5-dCTP (PerkinElmer) labeled cDNA in a reaction using Invitrogen Superscript III according to the manufacturer's instructions. cDNA was purified using a PCR purification kit (Qiagen), and concentrated in an Eppendorf Concentrator 5301 to a volume of 5 µl. For each *E. coli* K12 OciChip (Ocimum Biosolutions) one Cy3-labeled sample and one Cy5-labeled sample were hybridized. The cDNA mixture was heated at 95°C for 3 min in 100 µl hybridization buffer (Ocimum Biosolutions), and applied to the slides, which were hybridized for 16 h at 42°C. The slides were washed in decreasing salt concentrations, dried by centrifugation, and scanned with an Affymetrix 428 scanner. Two biological repeats of the experiment were carried out, each incorporating dye-swaps to account for differences in Cy dye incorporation, resulting in four technical repeats for each experiment. The datasets are deposited in the ArrayExpress database with the accession number E-TABM-876.

Data analysis was carried out using Imagene, version 5.1, and Genesight version 4. The mean values for each channel were log_2_-transformed and normalized using the LOWESS algorithm to remove intensity-dependent effects within the calculated values. Normalized values were used to calculate the Cy3∶Cy5 ratio from experimental and technical repeats with all values combined. Genes exhibiting greater than 2-fold change in abundance at one or more of the time points with a *p* value of ≤0.05 were deemed to be differentially regulated.

### Extra-cellular metabolite measurements

For overflow metabolite determination, a 1 ml sample removed from the chemostat at the times indicated was clarified by centrifugation (13000 *g* for 1 min) at 20°C. The cell-free supernatant fractions were analyzed in a volume of 500 µl containing 450 µl sample, 50 µl D_2_O and 1 mM trimethylsilylpropionate (TSP) as a standard. For intra-cellular samples, prior to NMR analysis, the pellet was resuspended in 500 µl D_2_O and TSP was added to a concentration of 100 µM. The samples were analyzed in 5 mm diameter tubes at 298 K. All spectra were acquired on a Bruker DRX-500 spectrometer operating at 500 MHz. The H_2_O signal was reduced by pre-saturation for 2 s applied during the recycle time. In the case of supernatant fractions, carbon decoupling was applied during acquisition to suppress ^13^C satellites. Spectra were processed and peaks quantified by integration using Topspin. Chemical shifts and concentrations were established by reference to TSP.

### Pyruvate dehydrogenase complex assays

Samples (20 ml) were removed from the chemostat at the indicated time points and immediately centrifuged at 5000 *g* at 20°C for 5 min. The supernatant was aspirated off and the pellet immediately frozen at −20°C. The pellet was defrosted and resuspended in 1 ml of 40 mM sodium phosphate buffer pH 7.4. The sample was sonicated then centrifuged at 13000 *g* for 5 min. The supernatant was kept on ice and the protein concentration was determined (BioRad protein assay kit). The extracts were assayed in a 1 ml volume containing 120 mM Tris buffer pH 8.5, 4 mM L-cysteine, 0.08 mM Coenzyme A, 0.2 mM thiamine pyrophosphate, 1.5 mM 3-acetylpyridine adenine dinucleotide, 3 mM MgCl_2_ and the reaction was started by adding 7.5 mM sodium pyruvate. Absorbance was measured against a pyruvate-free blank at 366 nm over 2 min using a Unicam UV Vis spectrophotometer.

### Western blot analysis

The optical density at 600 nm was determined for samples removed from the chemostat and a volume containing an equivalent cell density was harvested for each time point. The pellets were boiled in reducing Laemmli sample buffer before electrophoresis on sodium dodecylsulfate-polyacrylamide gels [25] followed by electroblotting onto Hybond-C membrane (Amersham). Proteins were detected using primary antibody raised in rabbit (1∶10000) followed by anti-rabbit immunoglobulin-horseradish peroxidase conjugate (1∶10000 dilution) (Amersham). Bound antibody was visualized by chemiluminescence (ECL detection kit, Amersham).
